# Neuronal density in the brain cortex and hippocampus in Clsnt2-KO mouse strain modeling autistic spectrum disorder

**DOI:** 10.18699/VJGB-22-44

**Published:** 2022-07

**Authors:** I.N. Rozhkova, S.V. Okotrub, E.Yu. Brusentsev, E.E. Uldanova, E.А. Chuyko, T.V. Lipina, T.G. Amstislavskaya, S.Ya. Amstislavsky

**Affiliations:** Institute of Cytology and Genetics of the Siberian Branch of the Russian Academy of Sciences, Novosibirsk, Russia; Institute of Cytology and Genetics of the Siberian Branch of the Russian Academy of Sciences, Novosibirsk, Russia Novosibirsk State University, Novosibirsk, Russia; Institute of Cytology and Genetics of the Siberian Branch of the Russian Academy of Sciences, Novosibirsk, Russia; Institute of Cytology and Genetics of the Siberian Branch of the Russian Academy of Sciences, Novosibirsk, Russia; Institute of Cytology and Genetics of the Siberian Branch of the Russian Academy of Sciences, Novosibirsk, Russia Novosibirsk State University, Novosibirsk, Russia; University of Toronto, Toronto, Canada; Scientific Research Institute of Neurosciences and Medicine, Novosibirsk, Russia; Institute of Cytology and Genetics of the Siberian Branch of the Russian Academy of Sciences, Novosibirsk, Russia

**Keywords:** mice, calsyntenin-2, brain, neuronal density, prefrontal cortex, hippocampus, autism spectrum disorder, мыши, кальсинтенин-2, мозг, плотность нейронов, префронтальная кора, гиппокамп, расстройства аутистического спектра

## Abstract

Autistic spectrum disorders (ASD) represent conditions starting in childhood, which are characterized by diff iculties with social interaction and communication, as well as non-typical and stereotyping models of behavior. The mechanisms and the origin of these disorders are not yet understood and thus far there is a lack of prophylactic measures for these disorders. The current study aims to estimate neuronal density in the prefrontal cortex and four hippocampal subf ields, i. e. СA1, СA2, СA3, and DG in Clstn2-KO mice as a genetic model of ASD. In addition, the level of neurogenesis was measured in the DG area of the hippocampus. This mouse strain was obtained by a knockout of the calsinthenin-2 gene (Clsnt2) in C57BL/6J mice; the latter (wild type) was used as controls. To estimate neuronal density, serial sections were prepared on a cryotome for the above-mentioned brain structures with the subsequent immunohistochemical labeling and confocal microscopy; the neuronal marker (anti-NeuN) was used as the primary antibody. In addition, neurogenesis was estimated in the DG region of the hippocampus; for this purpose, a primary antibody against doublecortin (anti-DCX) was used. In all cases Goat anti-rabbit IgG was used as the secondary antibody. The density of neurons in the CA1 region of the hippocampus was lower in Clstn2-KO mice of both sexes as compared with controls. Moreover, in males of both strains, neuronal density in this region was lower as compared to females. Besides, the differences between males and females were revealed in two other hippocampal regions. In the CA2 region, a lower density of neurons was observed in males of both strains, and in the CA3 region, a lower density of neurons was also observed in males as compared to females but only in C57BL/6J mice. No difference between the studied groups was revealed in neurogenesis, nor was it in neuronal density in the prefrontal cortex or DG hippocampal region. Our new f indings indicate that calsyntenin-2 regulates neuronal hippocampal density in subf ield-specif ic manner, suggesting that the CA1 neuronal subpopulation may represent a cellular target for early-life preventive therapy of ASD.

## Abstract (ru)

Расстройства аутистического спектра (РАС) – это группа состояний, возникающих в детском возрасте, для которых характерны трудности с социальным взаимодействием и общением, a также нетипичные модели поведения и склонность к стереотипии. Механизмы возникновения этой группы расстройств до сих пор не вполне понятны, и, следовательно, отсутствуют соответствующие методы профилактики. Целью исследования была оценка плотности нейронов в медиальной префронтальной коре и четырех областях гиппокампа, а именно СA1, СA2, СA3 и зубчатой извилины (DG) у мышей линии Clstn2-KO, которая может выступать в качестве генетической модели РАС. Кроме того, охарактеризовали уровень нейрогенеза в области DG гиппокампа. Данная линия получена путем нокаута гена кальсинтенина-2 (Clstn2) на основе мышей линии С57BL/6J; последняя была использована в настоящем исследовании в качестве контроля. Для определения плотности нейронов изготавливали серийные срезы соответствующих областей мозга на криотоме с последующим иммуногистохимическим окрашиванием и конфокальной микроскопией, для чего использовали нейрональный маркер (anti-NeuN) в качестве первичного антитела. Наряду с этим в области DG гиппокампа оценивали нейрогенез, для чего проводили иммуногистохимическое окрашивание с применением антитела против даблкортина (anti-DCX). В обоих случаях в качестве вторичного антитела был Goat anti-rabbit IgG. Плотность нейронов в области гиппокампа СА1 была снижена как у самцов, так и самок мышей Clstn2-KO по сравнению с контролем; у самцов обеих линий плотность нейронов была ниже в этой области по сравнению с самками. Помимо этого, были обнаружены различия между самцами и самками в двух других областях гиппокампа: в области CA2 – у мышей обеих исследованных линий, а в области CA3 лишь у мышей C57BL/6J плотность нейронов была меньше у самцов по сравнению с самками. Различий между исследованными группами в уровне нейрогенеза, а также в плотности нейронов в префронтальной коре и области DG гиппокампа не обнаружено. Полученные результаты показывают, что нокаут по гену Clstn2 приводит к избирательному снижению плотности нейронов в CA1-области гиппокампа, что может представлять собой клеточную мишень для ранней профилактики и возможной терапии РАС.

## Introduction

Diagnosis and prevention of autism spectrum disorders (ASD) at an early age is very important and requires identification of a specific molecular cellular target. Despite some progress in this area, for example, the discovery of the Fragile X, SHANK3, CASPR2 genes as risk factors for ASD, the mechanisms of this group of disorders are still not fully understood and, therefore, there are no appropriate methods of their prevention. The main reason for this is that both genetic and environmental factors are involved in the pathogenesis of autism, including, for example, epigenetic modifications of the genome, chromosome remodeling, oxidative stress, and many others (Waye, Cheng, 2017).

The hippocampal regions (CA1, CA2, CA3 and dentate gyrus – DG) are involved in memory-related processes: CA1 is important for working memory (Newmark et al., 2013), while CA3, CA4 and DG are in the circuit of declarative memory (Coras et al., 2014), and CA2 is associated with episodic (Navratilova, Battaglia, 2015) and social (Hitti, Siegelbaum, 2014) memory.

Structural abnormalities of the hippocampus have been reported for many complex psychiatric disorders, including vascular dementia (Kim et al., 2015), Alzheimer’s disease (Thomson et al., 2004), and ASD (Bauman, Kemper, 2005; Varghese et al., 2017). There are indications that people with ASD also have alterations in the prefrontal cortex, in particular, some neurons in this brain area are changed (Courchesne et al., 2011; Varghese et al., 2017). It has also been discussed that people with ASD demonstrate impaired neurogenesis (Gilbert, Man, 2017).

Several studies in ASD patients identified mutations in genes encoding synaptic proteins, including those involved in the regulation of cell adhesion (Bakkaloglu et al., 2008; Morrow et al., 2008; Bourgeron, 2015). Calcintenins (Clstns) are transmembrane synaptic proteins that belong to the superfamily of cadherin cell adhesion molecules. There are three types of Clstns (Clstn-1, -2 and -3) which are expressed postsynaptically (Hintsch et al., 2002) and contribute differently to the balanced activity of excitatory and inhibitory neurons; an imbalance in these processes is characteristic of some patients with ASD (Yip et al., 2009).

The absence of Clstn2 specifically reduces the density of inhibitory parvalbumin interneurons in some brain areas, which is manifested as insufficient inhibitory, but not excitatory, synaptic transmission in the pyramidal neurons of the hippocampal CA1 region (Lipina et al., 2016). In addition, a change in synapse architectonics was found in Clstn2- KO mice in the medial prefrontal cortex and hippocampus (Ranneva et al., 2020). Moreover, calsintenin-2 knockout (Clstn2- KO) mice demonstrate signs characteristic for ASD, such as hyperactivity, stereotypy, insufficient spatial learning and memory, altered social behavior with impaired ultrasonic vocalization (Lipina et al., 2016; Ranneva et al., 2017; Klenova et al., 2021).

The density of neurons in the prefrontal cortex and hippocampus, as well as the level of neurogenesis in the brain, have not yet been studied on such a genetic model of ASD as Clstn2-KO mice. Thus, the aim of this study was to characterize neuronal density in the medial prefrontal cortex, as well as in CA1, CA2, CA3, and DG regions of the hippocampus; and to evaluate the level of neurogenesis in Clstn2-KO mice.

## Materials and methods

Experimental animals. The study used 12 homozygous males and 14 females of the Clstn2-KO mouse strain knockout for the Clstn2 gene, as well as 15 males and 15 females of C57BL/6J mice (control) at the age of three months. Animals were kept in 36 × 25 × 14 cm (length × width × height) cages with wood bedding. Males and females were kept individually in a conventional vivarium of the Scientific Research Institute of Neurosciences and Medicine (Novosibirsk, Russia), at 20–22 °C, 12 dark : 12 light cycle, with free access to dry granulated chew (“Chara”, Assortiment-Agro, Russia) and purified water. All studies were performed in accordance with the European Convention for the Protection of Vertebrate Animals Used for Experimental and Other Scientific Purposes

Intracardiac perfusion. At the age of three months, the animals were perfused with phosphate buffered saline (PBS) and 10 % paraformaldehyde solution; thereafter, the brain was removed and fixed in phosphate buffer containing 30 % sucrose and 5 % formalin at +4 °C. Subsequently, the brain was immersed in Tissue-Tek O.C.T. compound (Sakura Finetek, USA), frozen and stored at –70 °C.

Brain sectioning. Cryosections were made for the following areas of the brain: 1) medial prefrontal cortex (MPC) at a distance of 2.46–2.22 mm from the bregma; 2) hippocampus (regions CA1, CA2, CA3 and DG) at a distance of –1.46…–1.82 mm from the bregma. Sections of 10 μm thick were done on an HM550 OP cryotome (Thermo Fisher Scientific, USA) at –25 °C and placed on Superfrost Plus, Menzel- Glaser glass slides (Thermo Fisher Scientific).

Immunohistochemical (IHC) staining. The slices was stained according to kit manufacturer’s protocols with minor modifications. Brain sections were dehydrated before staining, followed by 5 min of rehydration in PBS. Then, after rehydration in 10 mM citrate buffer (pH = 9) at 95 °C in a TW-2.02 water bath (Elmi, Latvia), heat-induced epitope unmasking was performed. The sections were removed from the buffer and cooled to room temperature. Then, the samples were washed three times in PBS-Tween buffer: PBS with the addition of 0.1 % Tween-20 P9416-100ML (Merck, Germany). Protein Block ab64226 (Abcam, UK) was added to each section for 5 min as recommended by the manufacturer; excess liquid was removed.

After the procedures of washing and incubating with the Protein Block, the primary antibody was added to the slices and left overnight at +4 °C in a humid dark chamber. The antibody concentrations used were 1:750 and 1:750 for anti- NeuN ab177487 (Abcam) and anti-DCX ab18723 (Abcam), respectively. Then the sections were washed with PBS-Tween buffer, excess liquid was removed; thereafter, 50 μL of secondary antibody Goat anti-rabbit IgG H&L AF488 ab150077 (Abcam) at a concentration of 1:600 was added to the slices and left in a humid dark chamber for two hours at +4 °C. Then the samples were washed with PBS-Tween buffer, excess liquid was removed, and the samples were placed in ProLong, Glass AntifadeMountant, Thermo P36982 (Thermo Fisher Scientific).

Neuron density analysis. Using a confocal laser scanning microscope LSM 780 (Carl Zeiss, Germany), EC Plan-Neofluar 20x/0.50 (Carl Zeiss), digital photographs were obtained, which were used to evaluate the density of neurons labeled with antibodies (https://ckp.icgen.ru/ckpmabo). The number of labeled neurons was counted using ImageJ. The number of antibody-labeled neurons was counted in at least three sections per animal, then the average per these sections was counted and the average volume density (mm3) was calculated.

Statistical analysis. The analysis of the obtained results was carried out using the software Statistica v. 10.0 (StatSoft, Inc., USA). All the data were tested for normality using the Shapiro–Wilk W-test. Data are presented as mean ± standard error of the mean (M ± SEM) and analyzed by two-way analysis of variance (ANOVA) followed by Fisher’s multiple comparison. Differences at p < 0.05 were considered as statistically significant.

## Results

**Fig. 1.**
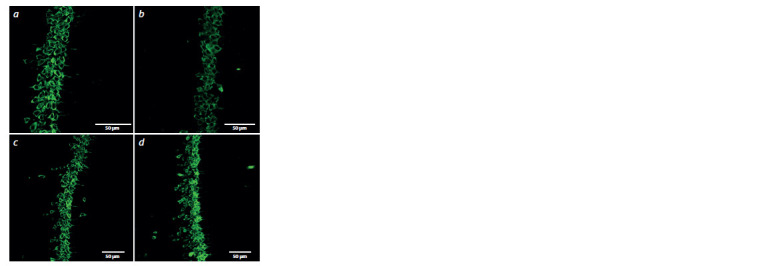
Micrographs of hippocampal CA1 region, neurons labeled with antibodies against the NeuN. a, b – females; c, d – males of the C57BL/6J (a, c) and Clstn2-KO (b, d ) strains.

Data on the density of neurons in the prefrontal cortex and hippocampus (CA1, CA2, CA3 and DG areas) are presented in the Table. A statistically significant effect on the density of neurons in the CA1 region of the hippocampus was found for the “sex” factor (F1,35 = 29.53, p <0.001) and the “group” factor (F2,35 = 16.68, p < 0.001), while there were no interactions of these factors (F1,35 < 1). Post-hoc comparison confirmed that both male ( p <0.001) and female ( p < 0.05) Clstn2-KO mice have fewer pyramidal neurons in the CA1 region of the hippocampus compared to control C57BL/6J (Fig. 1). Moreover, there were sex differences demonstrated: females of both strains had more pyramidal neurons ( p < 0.001) in the CA1 region of the hippocampus compared to males (see Fig. 1).

**Table 1.**
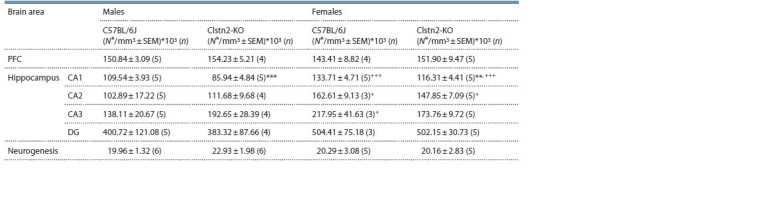
Neuronal density in hippocampus and prefrontal cortex; neurogenesis in dentate gyrus of the hippocampus Notе. N* – number of neurons in the region of interest; SEM – standard error of the mean; n – number of animals; ** p < 0.05 as compared with C57BL/6J of the same sex; ***p <0.001 as compared with C57BL/6J of the same sex; +p < 0.05 as compared with males of the same strain; +++p < 0.001 as compared with males of the same strain.

**Fig. 2.**
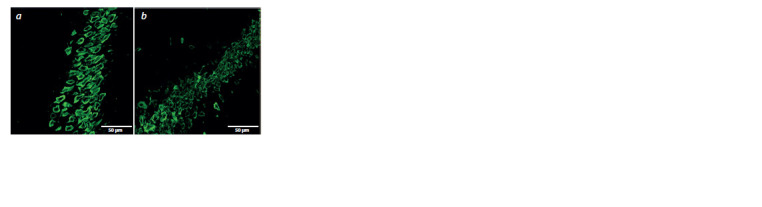
Micrographs of the hippocampal CA2 region. Neurons labeled with antibodies against NeuN in females (a) and males (b) of the Clstn2-KO strain.

The influence of the “sex” factor on the density of neurons in the CA2 region of the hippocampus was found (F1,31 = 12.03, p < 0.05). At the same time, the influence of the “group” factor (F1,31 <1) and the interaction of these factors (F1,31 < 1) were not found for the density of neurons in the CA2 region. Post-hoc comparison showed that females of both strains had more pyramidal neurons ( p < 0.05) in the CA2 region of the hippocampus compared to males (Fig. 2). However, there were no interstrain differences in the density of neurons in this region.

No influence of the factors “sex” (F1,35 = 1.66, p > 0.05) and “group” (F1,35 < 1) on the density of neurons in the CA3 region of the hippocampus was found, but the interaction of these factors was revealed (F1,35 = 4.36, p < 0.05). Post-hoc comparison confirmed that C57BL/6J females had more pyramidal neurons than males of this strain ( p <0.05).

To test the possibility that one of the reasons for the decrease in the density of neurons in the CA1 region in Clstn2-KO mice may be caused by the altered level of neurogenesis, its evaluation was carried out in the DG region of the hippocampus. Hippocampal neurogenesis was not influenced by the factors “sex” (F1,35 = 1.27, p > 0.05), and “group” (F1,35 < 1), and there was no interaction of these factors (F1,35 <1). Post-hoc comparison confirmed that there are no interstrain and sex differences in the level of neurogenesis in the DG region (see the Table).

## Discussion

In this study, no changes in the density of neurons in the prefrontal cortex were found in both male and female Clstn2- KO mice, although there is an alteration in neuronal density in this area of the brain in people with ASD (Courchesne et al., 2011). It should be noted, however, that human data are obtained by post mortem brain biopsy and should be discussed with caution, since only a few brain samples have been studied. In BTBR mouse strain, a model of idiopathic autism, no changes were found in the number of neurons in the prefrontal cortex (Stephenson et al., 2011), which is consistent with the results of our study in Clstn2-KO mice. However, other studies in BTBR mice have shown lower levels of extracellular acetylcholine and more kynurenic acid, but not serotonin, in this area of the brain (McTighe et al., 2013; Guo, Commons, 2017). It can be assumed that the development of ASD is associated with an imbalance of neurotransmitter systems in the prefrontal cortex, and the density of neurons in this structure is not a universal marker of these disorders.

In our current study, both male and female Clstn2-KO mice were found to have a reduced neuronal density in the CA1 region of the hippocampus. An earlier study had demonstrated a deficiency of inhibitory GABAergic neurons in the CA1 and CA3 regions of the hippocampus in mice of this strain (Lipina et al., 2016). We suppose that the decrease in the density of neurons in the CA1 region of the hippocampus revealed in Clstn2-KO mice in the current study is associated, among other things, with the detected decrease in GABAergic neurons mentioned above.

Structural studies using MRI have revealed a decrease in the relative volume of the hippocampus in patients with ASD aged 4 to 18 years (Sussman et al., 2015). Moreover, a change in the size of the hippocampus has also been found in adult patients with ASD (Braden et al., 2017). According to the results of the brain post mortem biopsy of people that suffered from ASD symptoms, changes in the neuronal density in certain areas of the hippocampus were found, the most pronounced changes were observed in the CA1 region (Lawrence et al., 2010; Greco et al., 2011).

Studies on various laboratory models also indicate the abnormalities in the CA1 region of the hippocampus are associated with the development of ASD. In particular, heterozygous mice deficient in the Tcf4 transcription factor, which demonstrate features of autism, showed an increased synaptic transmission in the CA1 region of the hippocampus (Kennedy et al., 2016). BTBR T+ tf/J mice exhibit behavior characteristic for ASD, accompanied by loss of neurons in the CA1 region of the hippocampus (Zhang et al., 2019). Heterozygous SHANK-3 mice, which are a well-known model of ASD, exhibited perforated synapses in the radial layer of the CA1 region of the hippocampus (Uppal et al., 2015), which confirms impaired synaptic plasticity in this region of the brain (Moessner et al., 2007). Fragile X chromosome syndrome, one of the forms of ASD associated with a disruption of the Fmr1 gene, also possessed specific changes in the pyramidal neurons of the CA1 region of the hippocampus (Sawicka et al., 2019). Thus, both the results of the current study and the results of the above mentioned studies in other mouse strains indicate that disorders in the CA1 region of the hippocampus can be considered as a specific marker of ASD

In our study, the density of neurons in Clstn2-KO females in the CA1 and CA2 regions of the hippocampus was higher than in males. Moreover, in our work, in female mice of the control strain C57BL/6J, the number of neurons in these areas, as well as in the CA3 region of the hippocampus, was also higher than in males, which may be a physiological feature non-related to ASD, despite the fact that, in humans, ASD is more common in boys than in girls in a ratio of 4.3:1 (Fombonne, 2003).

Disruption of the formation of new neurons in adulthood plays a significant role in the development of mental disorders (Schoenfeld, Cameron, 2015). Moreover, BTBR mice have been shown to have a significant impairment in adult neurogenesis (Stephenson et al., 2011). However, according to our data, neurogenesis in adult Clstn2-KO mice is not impaired. It is possible that the decrease in the density of neurons in the CA1 region found in Clstn2-KO mice is due to an increase in neurodegenerative processes, which, in particular, can lead to an imbalance in inhibitory and excitatory neurons

## Conclusion

A decrease in the density of neurons in the CA1 region of the hippocampus was found in both male and female Clstn2-KO mice compared to control C57BL/6J mice. Meanwhile, in Clstn2-KO mice, no changes in neuronal density were found in other areas of the hippocampus, and in the prefrontal cortex, the level of hippocampal neurogenesis was unaltered as well. The decrease in the density of neurons in the CA1 region of the hippocampus in Clstn2-KO mice can be considered as a specific characteristic for this model of autism spectrum disorders.

## Conflict of interest

The authors declare no conflict of interest.
